# Supplementary material for: Composition and Bioactivity of Chlorogenic Acids in Vegetable and Conventional Sweet Potato Vine Tips
Source: Foods. 2023 Oct 25;12(21):3910. doi: 10.3390/foods12213910 (PMC10649122; doi:10.3390/foods12213910)
Supplement: Supplementary file 1 [file foods-12-03910-s001.zip › foods-2640769-supplementary.pdf]

Composition and bioactivity of chlorogenic acids in vegetable and conventional sweet potato vine tips

Fantong Meng [1,†], Wantong Du [1,†], Yaxing Zhu [1], Ximeng Du [1], Chengchuang Song [1], Xi Chen [1], Xingtang Fang [1], Qinghe Cao [1,2], Daifu Ma [1,2], Yanhong Wang [1] and Chunlei Zhang [1,*]

1. Jiangsu Key Laboratory of Phylogenomics and Comparative Genomics, Institute of Cellular and Molecular Biology, College of Life Science, Jiangsu Normal University, Xuzhou 221116, China
2. Sweetpotato Research Institute, Chinese Academy of Agricultural Sciences, Xuzhou 221004, China

Corresponding author:

* Chunlei Zhang, E-mail: clzhang@jsnu.edu.cn

† These authors contributed equally to this paper.

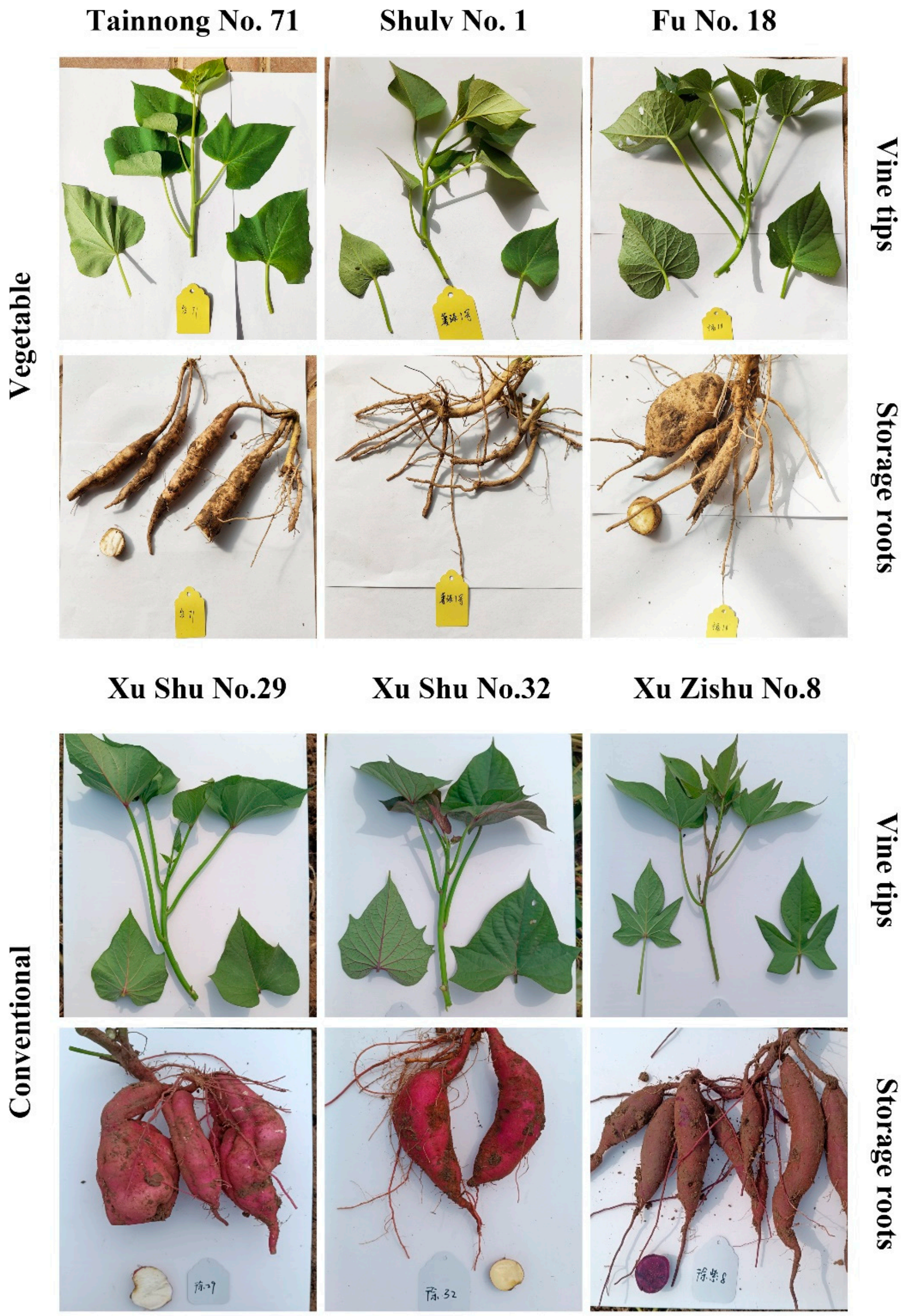

Figure S1. Vegetable and conventional sweet potato vine tips standard form.

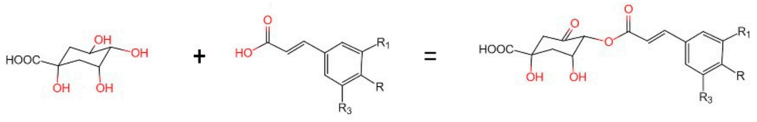

Quinic acid **Cinnamic acid**

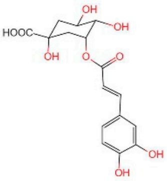

3-CQA

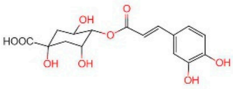

4-CQA

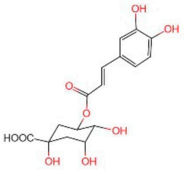

5-CQA

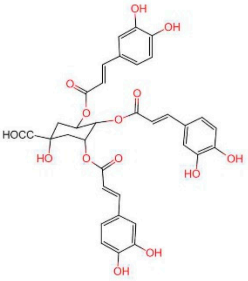

3,4,5-CQA

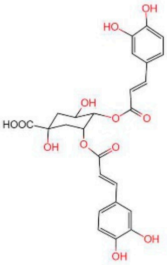

3,4-CQA

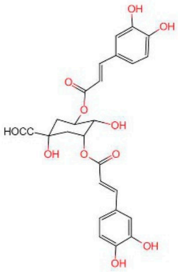

3,5-CQA

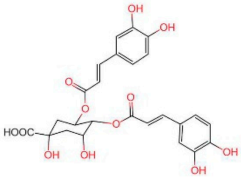

4,5-CQA

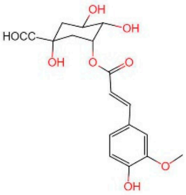

3-FQA

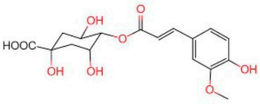

4-FQA

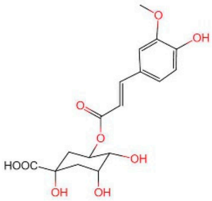

5-FQA

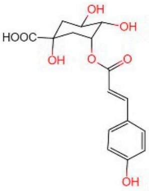

3-pCoQA

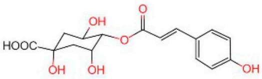

4-pCoQA

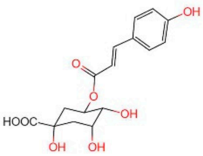

5-pCoQA

Figure S2. Structures of sweet potato chlorogenic acids with IUPAC nomenclature.

Table S1. The regressive equations and correlation coefficient of 12 CGAs

No.	CGA Name	Regression equation	R^2
1	3-O-caffeoylquinic acid	y = 112.16x - 488.95	0.9952
2	4-O-caffeoylquinic acid	y = 16.651x + 10.926	0.9999
3	5-O-caffeoylquinic acid	y = 99.018x - 99.2	1
4	3,4-di-O-caffeoylquinic acid	y = 99.431x - 336.76	0.9999
5	3,5-di-O-caffeoylquinic acid	y = 122.03x - 369.68	0.9999
6	4,5-di-O-caffeoylquinic acid	y = 73.509x - 173.87	1
7	3-O-feruloylquinic acid	y = 97.018x - 89.22	0.9897
8	4-O-feruloylquinic acid	y = 79.34x - 64.21	0.9954
9	5-O-feruloylquinic acid	y = 111.08x - 99.2	0.9827
10	3-O-p-coumaroylquinic acid	y = 98.018x - 99.32	0.9991
11	4-O-p-coumaroylquinic acid	y = 89.83x - 89.57	0.9987
12	5-O-p-coumaroylquinic acid	y = 94.48x - 94.62	0.9851

Y: Chromatographic peak area. X: CGA concentration (μg/mL:)

Table S2. Standard chlorogenic acid source.

CGA Name	Serial number	Manufacturer
3-O-caffeoylquinic acid	HY-N0055	MedChemExpress
4-O-caffeoylquinic acid	HY-N0787	MedChemExpress
5-O-caffeoylquinic acid	HY-N0722	MedChemExpress
3,4-di-O-caffeoylquinic acid	HY-N0057	MedChemExpress
3,5-di-O-caffeoylquinic acid	HY-N0056	MedChemExpress
4,5-di-O-caffeoylquinic acid	HY-N0058	MedChemExpress
3-O-feruloylquinic acid	HY-N6599	MedChemExpress
4-O-feruloylquinic acid	HY-N6598	MedChemExpress
5-O-feruloylquinic acid	HY-N7929	MedChemExpress
3-O-p-coumaroylquinic acid	TN7048	TargetMol
4-O-p-coumaroylquinic acid	T124698	TargetMol
5-O-p-coumaroylquinic acid	HY-N10543	MedChemExpress

Table S3. Mean, minimum, maximum, skewness and kurtosis values of the vegetable and normal sweet potato vine tips CGA.

	vegetable	conventional	vegetable	conventional	vegetable	conventional
**CQA**	3-CQA		4-CQA		5-CQA	
**Mean ± SD**	2.34±0.33	2.21±0.32	24.57±6.74	28.05±4.41	2.95±0.67	2.52±0.90
**Minimum**	1.8	1.79	15.22	23.21	2.22	1.16
**Maximum**	2.83	2.58	36.41	39.33	4.85	4.95
**Skewness**	0.23	-0.19	0.67	1.59	2.38	1.77
**Kurtosis**	1.13	1.83	-0.81	3.23	6.76	5.28
**DiCQA**	3,4-diCQA		3,5-diCQA		4,5-diCQA	
**Mean ± SD**	4.58±1.87	2.64±0.94	11.81±3.83	2.69±1.15	1.98±0.80	0.87±0.38
**Minimum**	1.84	1.01	5.93	1.03	0.99	0.31
**Maximum**	7.75	4.33	17.6	4.67	3.71	1.47
**Skewness**	0.2	-0.2	0.18	0.58	0.74	0.45
**Kurtosis**	0.9	0.11	1.02	0.87	0.48	1.09
**FQA**	3-FQA		4-FQA		5-FQA	
**Mean ± SD**	0.53±0.14	1.00±0.21	2.39±0.87	5.11±1.52	0.59±0.15	0.92±0.12
**Minimum**	0.35	0.74	1.31	3.9	0.36	0.78
**Maximum**	0.75	1.45	3.68	7.97	0.8	1.21
**Skewness**	-0.05	1.11	0.31	1.14	-0.26	1.26
**Kurtosis**	1.46	0.58	1.54	0.3	-1.43	1.87
**pCoQA**	3-pCoQA		4-pCoQA		5-pCoQA	
**Mean ± SD**	0.59±0.05	1.28±0.54	0.26±0.12	1.29±0.44	0.38±0.14	0.16±0.11
**Minimum**	0.5	0.79	0.15	0.87	0.16	0.05
**Maximum**	0.67	2.24	0.46	2.03	0.62	0.33
**Skewness**	-0.2	0.9	0.83	0.8	0.25	0.64
**Kurtosis**	0.83	0.72	1.19	1.16	0.62	1.48

Values (mg/g), Mean (n = 12).

Table S4. Sample material information summary.

Varieties	Name	Vine tip yield (t/hm^2)	Place of origin
	Shulv No. 1	25.78	Xuzhou, China
Vegetable sweet potato	Fu No. 18	39.03	Fujian, China
	Tainnong No. 71	25.55	Taiwan, China
	Xu Zishu No.8	20.12	Xuzhou, China
Conventional sweet potato	Xu Shu No.32	22.11	Xuzhou, China
	Xu Shu No.29	20.13	Xuzhou, China

Table S5. Pearson coefficients (P-values ($p < 0.05$)) for comparison of CGA isoforms in vegetable and conventional sweet potato vine tips.

Pearson Coefficients

**vegetable**	**3-CQA**	**4-CQA**	**5-CQA**	**3,4-CQA**	**3,5-CQA**	**4,5-CQA**	**3-FQA**	**4-FQA**	**5-FQA**	**3-pCoQA**	**4-pCoQA**	**5-pCoQA**
3-CQA	1							-0.6897	-0.6053		-0.7797	
4-CQA		1		-0.6302	-0.649						-0.594	-0.8742
5-CQA			1									
3,4-CQA		-0.6302		1	0.5894		0.6537	0.6807	0.7552			
3,5-CQA		-0.649		0.5894	1			0.7376	0.5773		0.831	0.7313
4,5-CQA						1				0.6914		
3-FQA				0.6537			1	0.6746	0.6795	-0.722		
4-FQA	-0.6897			0.6807	0.7376		0.6746	1	0.9124		0.8588	
5-FQA	-0.6053			0.7552	0.5773		0.6795	0.9124	1		0.7016	
3-pCoQA						0.6914	-0.722			1		
4-pCoQA	-0.7797	-0.594			0.831			0.8588	0.7016		1	0.784
5-pCoQA		-0.8742			0.7313						0.784	1

Pearson Coefficients

**conventional**	**3-CQA**	**4-CQA**	**5-CQA**	**3,4-CQA**	**3,5-CQA**	**4,5-CQA**	**3-FQA**	**4-FQA**	**5-FQA**	**3-pCoQA**	**4-pCoQA**	**5-pCoQA**
3-CQA	1	0.6232	0.6856		-0.7622	-0.8346	0.6889			0.6216		0.7353
4-CQA	0.6232	1					0.5766					
5-CQA	0.6856		1		-0.5562				0.7741			0.7016
3,4-CQA				1								
3,5-CQA	-0.7622		0.3094		1	0.7538		-0.5643	-0.677	-0.6073		-0.6913
4,5-CQA	-0.8346				0.7538	1						
3-FQA	0.6889	0.5766					1					
4-FQA					-0.5643			1		0.9596		0.9157
5-FQA			0.5993		-0.677				1			0.6697
3-pCoQA	0.6216				-0.6073			0.9596		1		0.9254
4-pCoQA											1	
5-pCoQA	0.7353		0.4922		-0.6913			0.9157	0.6697	0.9254		1
